# No Influence of Dabigatran Anticoagulation on Hemorrhagic Transformation in an Experimental Model of Ischemic Stroke

**DOI:** 10.1371/journal.pone.0040804

**Published:** 2012-07-24

**Authors:** Ferdinand Bohmann, Ana Mirceska, Josef Pfeilschifter, Edelgard Lindhoff-Last, Helmuth Steinmetz, Christian Foerch, Waltraud Pfeilschifter

**Affiliations:** 1 Department of Neurology, University Hospital, Goethe University, Frankfurt am Main, Germany; 2 Department of General Pharmacology and Toxicology, University Hospital, Goethe University, Frankfurt am Main, Germany; 3 Department of Internal Medicine, Division of Vascular Medicine, University Hospital, Goethe University, Frankfurt am Main, Germany; Julius-Maximilians-Universität Würzburg, Germany

## Abstract

**Background:**

Dabigatran etexilate (DE) is a new oral direct thrombin inhibitor. Clinical trials point towards a favourable risk-to-benefit profile of DE compared to warfarin. In this study, we evaluated whether hemorrhagic transformation (HT) occurs after experimental stroke under DE treatment as we have shown for warfarin.

**Methods:**

44 male C57BL/6 mice were pretreated orally with 37.5 mg/kg DE, 75 mg/kg DE or saline and diluted thrombin time (dTT) and DE plasma concentrations were monitored. Ischemic stroke was induced by transient middle cerebral artery occlusion (tMCAO) for 1 h or 3 h. We assessed functional outcome and HT blood volume 24 h and 72 h after tMCAO.

**Results:**

After 1 h tMCAO, HT blood volume did not differ significantly between mice pretreated with DE 37.5 mg/kg and controls (1.5±0.5 µl vs. 1.8±0.5 µl, p>0.05). After 3 h tMCAO, DE-anticoagulated mice did also not show an increase in HT, neither at the dose of 37.5 mg/kg equivalent to anticoagulant treatment in the therapeutic range (1.3±0.9 µl vs. control 2.3±0.5 µl, p>0.05) nor at 75 mg/kg, clearly representing supratherapeutic anticoagulation (1.8±0.8 µl, p>0.05). Furthermore, no significant increase in HT under continued anticoagulation with DE 75 mg/kg could be found at 72 h after tMCAO for 1 h (1.7±0.9 µl vs. control 1.6±0.4 µl, p>0.05).

**Conclusion:**

Our experimental data suggest that DE does not significantly increase hemorrhagic transformation after transient focal cerebral ischemia in mice. From a translational viewpoint, this indicates that a continuation of DE anticoagulation in case of an ischemic stroke might be safe, but clearly, clinical data on this question are warranted.

## Introduction

Atrial fibrillation (AF) is a severe independent risk factor of stroke, its attributable risk increasing with age up to more than 20% [1]. INR-driven oral anticoagulation with vitamin K antagonists to an INR of 2–3 reduces the risk of an ischemic stroke by over 60% [2] and has been the standard of stroke prevention in patients with AF for over 50 years. In the Randomized Evaluation of Long-Term Anticoagulation Therapy (RE-LY) trial, two fixed-dose regimens of dabigatran etexilate (DE) (110 mg or 150 mg bid) showed a superior risk-to-benefit ratio in comparison to warfarin in patients with AF for primary and secondary prevention of stroke [3]. Especially striking was the risk reduction of intracerebral hemorrhage in both DE dose groups compared to warfarin. The RE-LY trial establishes DE as an alternative to warfarin as an anticoagulant for stroke prevention in patients with AF.

DE is an orally administered prodrug which is rapidly converted by a serum esterase into its active form dabigatran. As a potent, competitive and reversible direct thrombin inhibitor, DE reaches maximum plasma concentrations within 2 hours after oral administration [4]. It has an estimated half-life time from 12 to 17 hours and 80% are excreted via the kidneys. DE does not require frequent coagulation monitoring like warfarin and has a low risk of drug-drug and food-drug interactions. Its predictable pharmacokinetic profile allows an effective oral anticoagulation with a fixed-dose regimen [5]. The anticoagulatory effect of DE is not fully assessed by routine coagulation parameters. While thrombin clotting time (TT), and activated partial thromboplastin time (aPTT) are altered by DE, prothrombin time (PT, INR) is not a useful parameter to evaluate anticoagulant activity of DE [6].

Even under optimal oral anticoagulation, patients with AF still remain at a residual risk of ischemic stroke. In the RE-LY trial, the risk of ischemic stroke was 1.34%/yr in the DE 110 mg bid group, 0.92%/yr in the 150 mg DE bid group and 1.2%/yr in the warfarin group [3]. Current guidelines do not recommend anticoagulation in acute cardioembolic stroke and in clinical practice; warfarin is discontinued in patients with an acute stroke. We have previously shown that warfarin pretreatment leads to an excessive hemorrhagic transformation (HT) in mice after tMCAO [7]. So far, no data on the HT risk after stroke under DE anticoagulation are available.

The aim of this study was to examine the influence of dabigatran anticoagulation on hemorrhagic transformation and neurological outcome in an experimental model of ischemic stroke in mice.

## Methods

### Animals

We used male C57BL/6 mice (strain J, 8–10 weeks, mean 25.5 g range 22.7–28.1 g, Janvier, Le Genest Saint Isle, France) according to the National Institute of Health Guide for the Care and Use of Laboratory Animals (NIH Publications No. 80–23, revised 1996). All experiments were approved by the local governmental authorities (Regierungspraesidium Darmstadt, approval number F 143/48). All animals received water and food without restrictions. All surgery was performed under isoflurane anesthesia and every effort was made to minimize suffering. ARRIVE guidelines were considered to rise the reproducibility and quality of our data [8].

### Sample Size Calculation and Study Design

Sample size calculation was based upon our previous study examining HT in vehicle or warfarin-treated mice subjected to the same stroke model [7]. In this study, an HT volume of 5.2±2.7 µL was observed in mice anticoagulated with warfarin to an international normalized ratio of 2.9±0.9 prior to 3 h tMCAO in comparison to an HT volume of 0.3±0.4 µL in controls who did not receive a pretreatment. To detect this difference with a power (1– β) of 0.8 and a level of acceptability of a false positive result (α) of 0.05, a sample size of 4 animals per group is required. Anticipating equal or smaller differences in HT between DE and control animals, we randomized six mice per group. For groups with an observation period of 72 h after tMCAO, we randomized seven mice per group to compensate for dead or excluded animals (Table 1).

**Table 1:** Inclusion – exclusion criteria for MCAO experiments.

Observation period	Experimental group	Included mice	Excluded mice	Died during observation period (Total autopsy)	Total mice			
			*mNSS <3 and lack of decrease in Doppler flow*	*Extracerebral* *hemorrhage*	*SAH*	*Died during operation*		
**24** **h**	MCAO 1 h in non-anticoagulatedmice (control)	5	0	0	1	0	1	6
	MCAO 1 h in DE-treated mice(37.5 mg/kg)	6	0	0	0	0	0	6
	MCAO 3 h in non-anticoagulatedmice (control)	6	0	0	0	0	2	6
	MCAO 3 h in DE-treated mice(37.5 mg/kg)	5	0	0	1	0	0	6
	MCAO 3 h in DE-treated mice(75 mg/kg)	5	0	0	1	0	0	6
**72** **h**	MCAO 1 h in non-anticoagulatedmice (control)	6	0	0	1	0	3	7
	MCAO 3 h in DE-treated mice(75 mg/kg)	5	0	0	1	1 (no hemorrhage)	3	7

**Figure 1:**
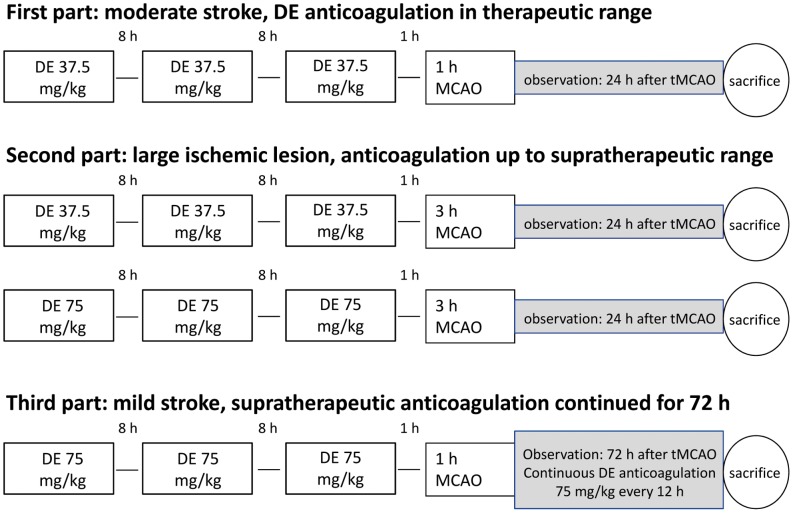
Timeline diagram of the experimental procedures.

First, we ascertained that our DE anticoagulation paradigm detailed in the following led to DE plasma concentrations that mirrored the therapeutic situation. In the first experiment, we randomized six mice per group to DE 37.5 mg/kg or saline to assess the influence of prior DE anticoagulation on HT volume and neurological deficit 24 h after the onset of 1 h tMCAO (Figure 1). Not finding significant differences in this model of moderate stroke, we randomized six mice per group in the second part of our study to DE 37.5 mg/kg, DE 75 mg/kg or saline prior to 3 h tMCAO. In order not to underestimate the risk of HT under DE anticoagulation, we doubled the DE dose in the second experiment to mimic the effect of supratherapeutic DE plasma concentrations that may occur in patients with reduced DE elimination, i.e. due to renal insufficiency. In the third part of our study, we randomized seven mice per group to either supratherapeutic DE anticoagulation (75 mg/kg) continued over an observation period of 72 h after 1 h tMCAO or saline. For this third part of our study, we chose an occlusion time of 1 h, because mice with an occlusion of 3 h had shown grave weight loss in the second part of our study as a sign of severe impairment, which might have led to inacceptably high rates of death and exclusion.

### Exclusion Criteria

Exclusion criteria were a missing cerebral blood flow decrease over the right MCA and/or a missing relevant neurological deficit (mNSS <3) as signs of an inadequate MCA occlusion. Massive bleeding during surgery or death not related to cerebral ischemia also resulted in exclusion. Therefore, every mouse which died within 24 h received a complete autopsy for exclusion of extracranial sources of hemorrhage. Mice with a subarachnoid hemorrhage (SAH) were also excluded after a complete autopsy. A weight loss of more than 20% as a sign of severe impairment of the mice also led to sacrifice and exclusion (Table 1).

### tMCAO, Blinding and Randomization

Transient middle cerebral artery occlusion (tMCAO) for 1 or 3 h was performed as described previously [6]. All surgery was performed in the afternoon under anesthesia with 1.5% isoflurane (Forene™; Abbott, Wiesbaden, Germany) under spontaneous respiration and 0.1 mg/kg buprenorphine for analgesia (Temgesic™; Essex Pharma, Munich, Germany). The depth of anesthesia was tested with the flexor reflex. Focal cerebral ischemia was induced by inserting standardized monofilaments with a tip diameter of 0.23 mm into the right MCA, which lead to highly reproducible ischemic lesion sizes between 5–10% of the respective mean values [9] (Doccol, Redlands, CA, USA). Regional cerebral blood flow was constantly monitored by laser Doppler flowmetry (PF5010, Perimed, Sweden) to confirm vessel occlusion. Following the operation, all animals received regular drinking water and food without restrictions. The operator was blinded to the anticoagulant pretreatment and mice were randomly assigned to treatment group and sequential order of operation by a computer generated list [10]. Besides anticoagulation all groups were treated equally. Neurological deficit and hemoglobin assays were performed in a blinded fashion.

### Evaluation of Ischemic Lesion Size

As a pretest for determination of ischemic lesion size after different tMCAO occlusion times, three groups were evaluated after 1 h, 2 h or 3 h tMCAO (n = 3 per group). We stained freshly prepared brain slices of 1 mm thickness with 2% 2,3,5-triphenyltetrazolium chloride (TTC, Merck KgaA, Darmstadt, Germany) that stains vital mitochondria and performed planimetry of the ischemic lesion (white), the contra- and the ipsilesional hemisphere with the National Institutes of Health Image J software. Lesion size was corrected for edema by multiplying the infarct volume by the ratio of the contralateral to the ipsilateral hemisphere volume.

### Oral Anticoagulation with Dabigatran Etexilate

The experimental groups received DE (Pradaxa™, Boehringer Ingelheim, Ingelheim, Germany). 110 mg tablets were freshly dissolved in 5 ml (22 mg/ml) or 10 ml (11 mg/ml) saline solution. DE was dosed depending on the target dose (11 mg/ml for 37.5 mg/kg and 22 mg/ml for 75 mg DE/kg) and the body weight of the mouse. Under a short isoflurane anesthesia 3.4 µl/g body weight were administered via a gastric tube. Mice were fed three times with 8 h intervals, the last oral administration was 1 hour before tMCAO or coagulation monitoring, respectively. For groups with an observation period of 72 h we performed a maintenance dose every 12 h after surgery. Oral gavages of comparable dosages were shown to result in significant aPTT prolongation in rats [11]. Linear dose-dependent aPTT prolongation was also demonstrated by our group in a murine model for intracranial hemorrhage in mice [12]. Control mice received equal volumes of saline solution.

DE concentrations were determined by measuring thrombin time in diluted samples (dTT) with the Hemoclot™ test based on the inhibition of a defined amount of human thrombin (Hyphen BioMed, Neuville-sur-Oise, France) which enables quantitative measurement of DTI activity in plasma. 450 or 900 µl mouse blood was drawn into 0.109 mol/l tri-sodium citrate in a 9∶1 ratio and plasma was obtained by centrifugation (15 min 2500 g at 15°C). Mouse plasma was diluted 1∶8 with factor diluent and mixed with two parts of normal pooled human plasma (50 µl prediluted mouse plasma and 100 µl normal pooled human plasma). Clotting was initiated by adding a constant amount of highly purified human α-thrombin and clotting time was measured on a calibration curve by using commercially available dabigatran calibrators. A direct linear relationship between dabigatran concentrations and clotting time has been shown for TT values from 30 to 75 seconds [6].

### HT Volume Determination

After 24 h, mice were transcardially perfused with 30 ml PBS under deep isoflurane anesthesia. Hemoglobin concentration was measured for each hemisphere separately following a previously described protocol [12]. Hemispheres were homogenized, subjected to ultrasound for 60 s and centrifuged (13.000 rpm, 4°C, 30 min) before photometric analysis of the supernatant mixed with Drabkin’s Reagent solution (one vial of the Drabkin’s Reagent, Sigma-Aldrich, Taufkirchen, Germany, with 1000 ml of water and 0.5 ml of Brij® 35 Solution, Product Code B 4184) in duplicates at 540 nm. HT volume was calculated based on a standard curve (data not shown) and the values of both hemispheres were added for the HT volume of the whole brain.

Mice found dead within the observation period could not be subjected to transcardial perfusion. In this case, we performed a total autopsy to exclude extracerebral bleeding and subarachnoid hemorrhage (SAH) (Table 1). Afterwards, we analyzed the non-perfused brains and subtracted 0.31 µl from the HT blood volume. This value was found to be the mean difference in HT blood volume between 5 unperfused and 5 perfused brains (data not shown).

### Neurological Deficit

Neurological deficit was assessed using the modified Neurological Severity Score (mNSS, modified from Chen [13]). The 14-point-mNSS includes testing hemiparesis, gait, coordination and sensory functions (Table S1). Pinna and corneal reflex were tested bilaterally. We assessed mice twice in videotaped sequences just before reperfusion and at the end of the observation period (Video S1). Every video sequence includes 60 s spontaneous motion activity. If mice stopped moving during the observation, they were stimulated by being raised up a few centimeters. We also videotaped two attempts of the hanging wire test. We placed the mice carefully on a bar of wood (8 mm diameter) 20 cm above the ground until they attained firm grip. The time period to falloff was recorded with a maximum of 60 seconds. The test was repeated two times. Mice were not trained before. The observation was performed by the surgeon in a blinded fashion. Mice that died within the observation period were given the maximum of 14 points in the mNSS functional outcome score.

### Statistical Analyses

Graph Pad Prism 4 (Graph Pad Software Inc., La Jolla, CA, USA) was used for statistical analysis. Results are given as mean ± SD and graphically presented as a box and whiskers plot depicting the median, extreme values and the 25–75 interquartile range. Statistical significance was assessed with a one-way ANOVA with Bonferroni’s correction and Bonferroni’s Multiple Comparison Test for HT values. Gaussian distribution was tested with the Kolmogorov-Smirnov P value directly, without the Dallall-Wilkinson-Lilliefor correction, well-knowing that the results for small sample sizes have to be interpreted very carefully. Neuroscore data are given as median and range and depicted in a dot plot. Statistical significance was assessed using a Mann-Whitney Test for two groups and a Kruskal-Wallis-Test with Dunn’s correction for three or more groups. For mice that died within the first 24 h, the maximum mNSS of 14 points was given to perform an intention-to-treat analysis.

## Results

### Ischemic Lesion Size

tMCAO for 1 h led to an ischemic lesion size of 44.5±6.5 mm^3^ (n = 3) after 24 h, for 2 h tMCAO it was 96.0 mm^3^ (n = 2, one mouse died during the operation) and for 3 h tMCAO it was 121.6±11.1 mm^3^ (n = 3) (data not shown).

### Anticoagulation Levels

**Figure 2:**
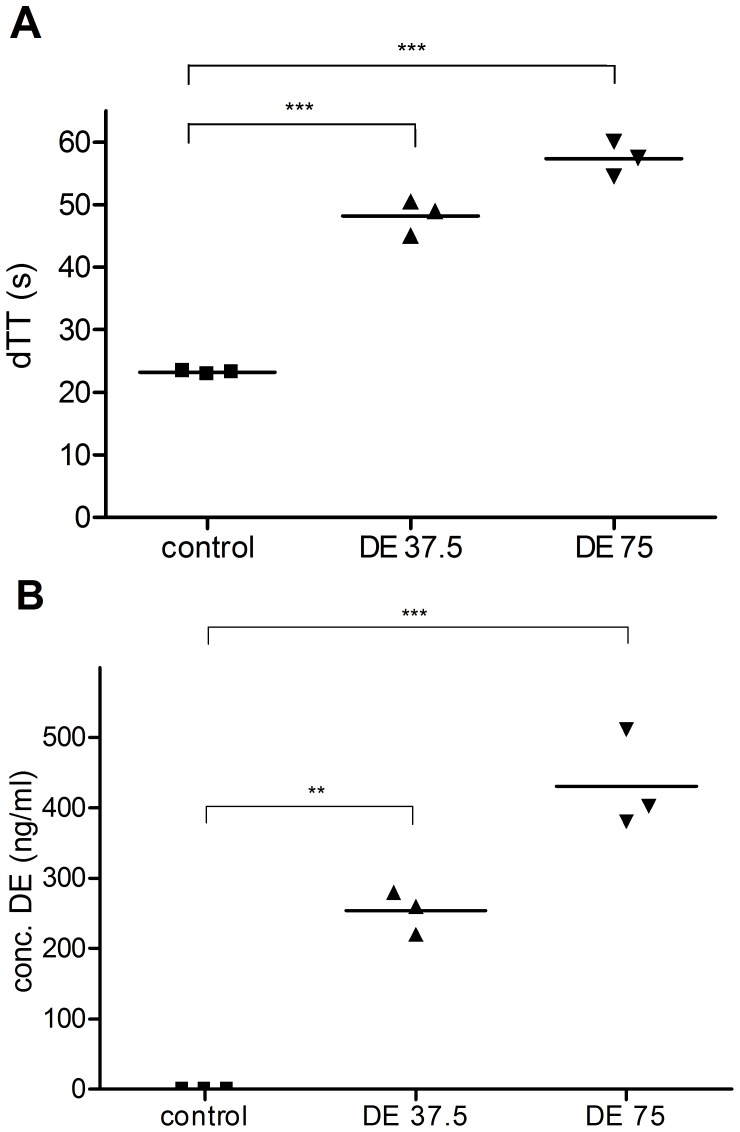
DE leads to a prolongation of the dTT in linear correlation with DE plasma concentrations (Hemoclot™ assay). A) dTT was measured with the Hemoclot™ assay in anticoagulation-naïve mice (n = 4), mice receiving 3×37.5 mg/kg over a 24 h feeding period (n = 3) and mice receiving 3×75 mg/kg (n = 3). Statistical significance was assessed with one-way ANOVA and Bonferroni correction. B) Calibration of the coagulometer with lyophilized standard DE plasma gives the DE concentration from the dTT values. Statistical significance was assessed with one-way ANOVA and Bonferroni correction. ** p<0.01; *** p<0.001.

Oral anticoagulation with DE at doses of 37.5 mg/kg and 75 mg/kg led to significant dTT prolongation (37.5 mg/kg: 48.2±2.8 s vs. 23.2±0.3 s in control mice; 75 mg/kg: 57.3±2.8 s;) (Figure 2A). This corresponds to DE plasma concentrations of 253.3±30.6 ng/ml in the group receiving 37.5 mg/kg and 431.1±69.8 ng/ml in the group receiving 75 mg/kg while saline-treated controls were at 0 ng/ml (Figure 2B). Mice with a continuation of anticoagulation over the observation period of 72 h showed DE plasma concentrations of 646.0 ng/ml and 767.2 ng/ml, whereas control mice were at 0 ng/ml (data not shown).

### DE Anticoagulation does not Lead to Higher HT Volumes

**Figure 3:**
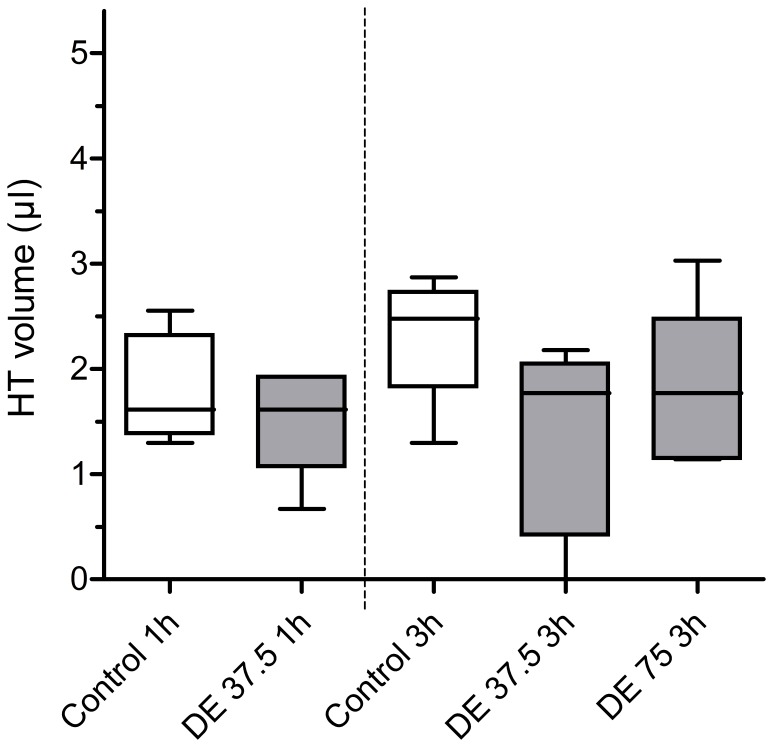
Mice pretreated with DE did not show a greater risk of HT after tMCAO than control mice. The left two bars show HT volume after 1 h MCAO in control mice (n = 5) and DE-pre-treated mice 37,5 mg/kg (n = 6). Shown on the right is the HT volume after 3 h MCAO in control mice (n = 6), DE-pre-treated mice 37,5 mg/kg (n = 5) and DE-pre-treated mice 75 mg/kg (n = 5). Haemoglobin assay was used for HT blood volume measurement and results are given in µl. Results are showed in a box and whiskers plot depicting mean values, extreme values and the 25 to 75 percent interquartile range. Statistical significance was assessed with a one-way ANOVA with Bonferroni correction. No significant differences were detected.

**Figure 4:**
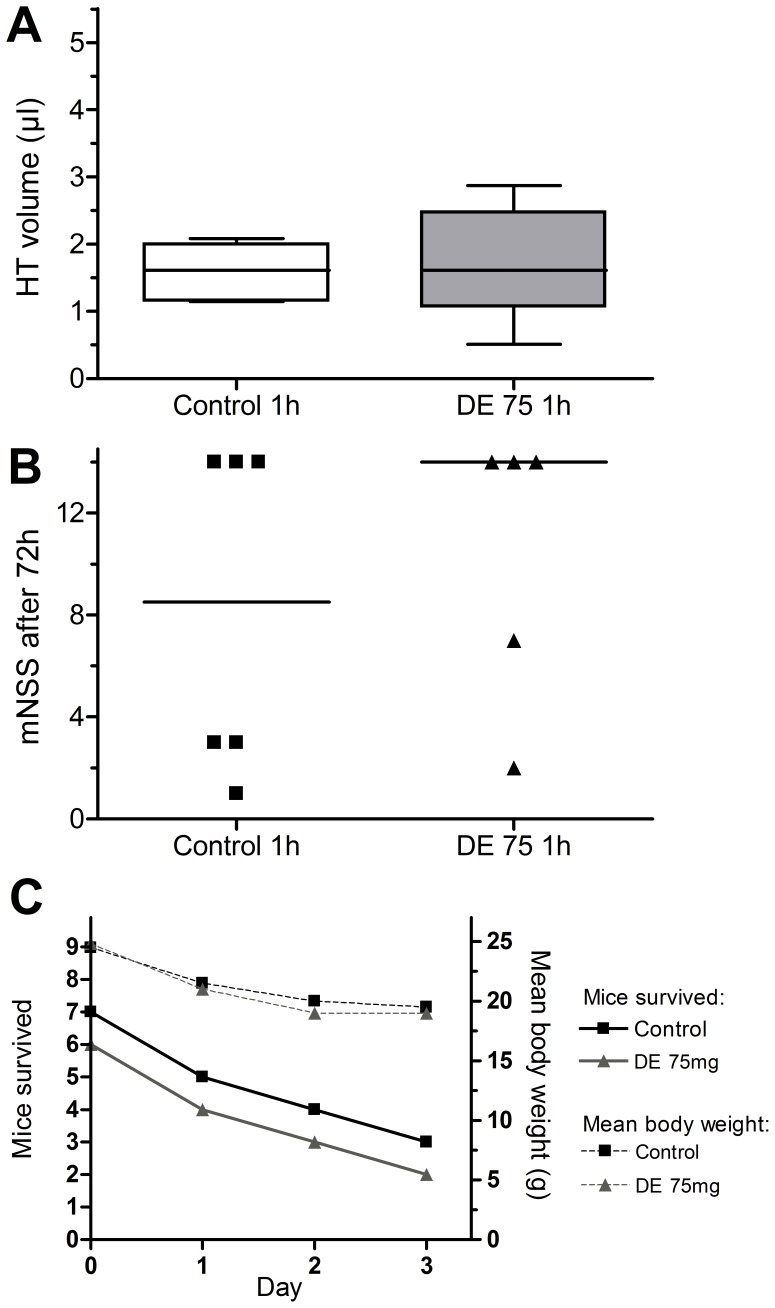
No difference in HT and neurological outcome under continuous anticoagulation 72 h after 1 h tMCAO. A) After 72 h of continuous anticoagulation after 1 h tMCAO, HT blood volume in DE 75 mg/kg pretreated mice (n = 6) and in control mice (n = 7) showed no significant difference. Haemoglobin assay was used for HT blood volume measurement. Results are given in µl presented in a box and whiskers plot depicting mean values, extreme values and the 25 to 75 percent interquartile range. B) Neurological function was evaluated on a 14 point scale (mNSS) after 72 h. Mice which died during the observation period were given 14 points as the worst outcome on the mNSS scale. The values of single mice and the medians are depicted in a dot plot. C) The number of surviving mice per group is given besides their mean body weight in gram.

Anticoagulation-naïve mice showed an HT blood volume of 1.5±0.5 µl 24 h after 1 h tMCAO. Pretreatment with DE (37.5 mg/kg) did not lead to a significant increase in HT blood volume (1.8±0.5 µl) (Figure 3). In the second part of our study evaluating HT 24 h after the onset of 3 h tMCAO, pretreatment with DE 37.5 mg/kg and even DE 75 mg/kg did also not lead to an increased degree of HT in comparison to non-anticoagulated mice (2.3±0.5 µl in control mice vs. 1.3±0.9 µl in DE 37.5 mg/kg-treated mice vs. 1.8±0.8 µl in DE 75 mg/kg-treated mice)(Figure 3). Even mice with DE anticoagulation that was continued at supratherapeutic drug levels for 72 h after tMCAO did not show a significant increase in HT volume compared to control mice (1.6±0.4 µl in control mice vs. 1.7±0.9 µl in DE 75 mg/kg) (Figure 4). All groups passed normality test (p>0.10).

### DE Pretreatment has no Influence on Neurological Outcome after Transient MCAO

**Figure 5:**
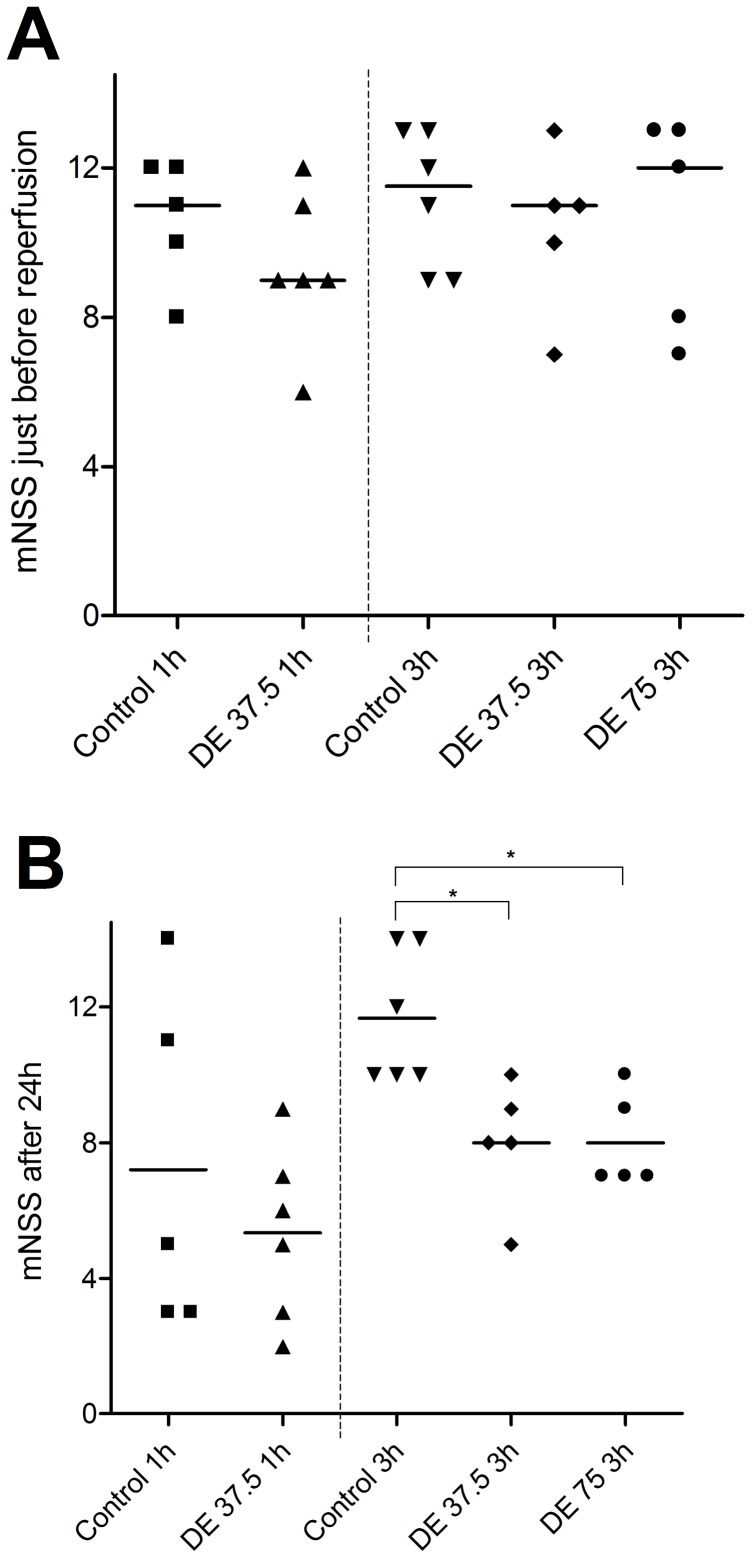
DE pretreatment does not lead to symptomatic hemorrhagic transformation after tMCAO. A) Neurological function after 1 h and 3 h tMCAO was assessed on a 14 point scale (mNSS) directly prior to reperfusion and at B) 24 h. The values of single mice and the medians are depicted in a dot plot. Statistical significance was assessed with a Mann-Whitney Test for two groups and a Kruskal-Wallis-Test with Dunn’s correction for three or more. * p<0.05.

In groups with an observation period of 24 h after tMCAO median mNSS values just before reperfusion after 1 h occlusion of the right MCA were 11 in non-anticoagulated mice (range 8–12) and 9 in DE-anticoagulated mice (range 6–12). During the observation period, both groups showed a functional improvement without significant differences after 24 h (control: 5, range 3–14 vs. DE 37,5 mg/kg: 6, range 2–9, p>0.05) (Figure 5).

After 3 h tMCAO, mNSS values directly before reperfusion did also not show significant differences (control: 12, range 9–13; DE 37.5 mg/kg: 11, range 7–13; DE 75 mg/kg: 12, range 7–13). 24 h after tMCAO mice showed a slight improvement of their functional neurological status in all three groups that was more pronounced in the DE-treated groups (control: 11 range 10–14; DE 37.5 mg/kg: 8 range 5–10; DE 75 mg/kg: 7 range 7–10) (Figure 5). Post-hoc power analysis, however, resulted in a statistical power clearly below 0.8, so a positive of effect of DE anticoagulation on functional outcome after MCAO cannot be postulated based on these data.

Mice who were continuously anticoagulated to supratherapeutic DE levels for 72 h following 1 h tMCAO did also not show a significant difference in functional neurological outcome compared to the control group (control: median 8.5, range 1–14; DE 75 mg/kg: median 14, range 2–14) (Figure 4). In comparison to the mNSS evaluation after 24 h, the surviving mice showed a distinct improvement 72 h after surgery. Nevertheless many mice died before they could reach neurological testing at 72 h, so these results have to be interpreted very carefully.

Weight loss was not statistically different between treatment groups after 24 h, neither after 1 h tMCAO nor with 3 h tMCAO (data not shown). One mouse in the control group (1 h MCAO) and two mice in the control group (3 h MCAO) died before evaluation 24 h after tMCAO induction, so they were given a maximal mNSS score of 14 points.

Mice which died in the two treatment groups with an observation period of 72 h were also graded 14 on the mNSS. Both long-term observation groups showed a considerable mortality within the observation period that was linked to weight loss and did not differ significantly between DE-treated mice and controls (Fig. 4).

## Discussion

In this study, we investigated whether pretreatment with the direct thrombin inhibitor DE had an influence on HT volume and functional outcome in an experimental model of ischemic stroke. We show that neither HT volume nor functional outcome at 24 h differ significantly between mice treated with DE or vehicle prior to 1 h and 3 h tMCAO. The lower dose of our study represents the therapeutic range measured in participants of clinical trials of DE taking 150 bid and the higher dose of our study represents supratherapeutic drug levels. Even mice who were continuously anticoagulated to supratherapeutic DE levels for 72 hours following tMCAO did not show an increase of HT compared to controls. This is in sharp contrast to our previous findings in mice undergoing MCAO under warfarin anticoagulation [7], which showed a 14- to 17-fold higher HT blood volume in the brains of warfarin-anticoagulated mice after tMCAO as compared to controls in mice anticoagulated to a mean INR of 1.9 and 2.9, respectively. Our findings indicate a superior safety profile of DE compared to warfarin in terms of HT after experimental stroke occurring under anticoagulation. The long-term data with continued DE anticoagulation over 72 h even show that it might be safe to continue dabigatran anticoagulation after a stroke under this treatment.

We used the well established stroke model of transient right middle cerebral artery occlusion with two different occlusion times [14]. The infarct sizes in our experimental model for 1 h, 2 h or 3 h occlusion time match infarct sizes obtained in previous measurements [7]. For the first part of our study we attempted to model a moderate territorial infarction without malignant brain swelling, so we chose an occlusion time of 1 h, leading to an average infarct volume of 44.5±6.5 mm^3^. To exclude the possibility of an underestimation of the HT risk due to small lesion sizes with little blood-brain barrier breakdown, we performed 3 h tMCAO with an average infarct volume of 121.6±11.1 mm^3^ in the second part of our study, well aware that such large ischemic lesions, mimicking malignant middle cerebral artery infarctions, represent only a small fraction of strokes commonly observed in human patients. However, this experimental paradigm was chosen because it leads to significant damage at the blood-brain barrier, including activation of matrix metalloproteinases, disintegration of cell-cell and cell-matrix junctions and endothelial cell dysfunction [15] and thus represents maximal ischemic tissue damage rendering the brains most vulnerable to HT. Several clinical studies, e.g. retrospective analyses of patient data entered into the National Institute of Neurological Disorders and Stroke rt-PA trial, identified ischemic lesion size [16] and stroke severity [17] as independent predictors of HT risk after stroke.

In previous studies, we established a model for oral anticoagulation with DE in mice [12]. We aimed at carefully controlling the anticoagulant activity by measuring the DE plasma concentration via the diluted thrombin time (dTT). Oral administration of DE via gavage for three times during 24 h resulted in stable and well-reproducible values in the Hemoclot™ assay. Consistent with data from humans [6], we have shown previously that prothrombin time (PTT) is less sensitive in detecting DE anticoagulant effects [12]. There is still a paucity of clinical data on DE plasma concentrations reached in humans with regular dabigatran intake. Pharmacokinetic modeling from the PETRO trial and the RE-LY trial [6], [18], [19] has shown mean peak plasma concentrations of 184 ng/ml (95% CI 64–443 ng/ml) and trough plasma concentrations of 90 ng/ml (95% CI 31–225 ng/ml) in trial patients administered 150 mg DE bid [6] which is the dose that is currently used for stroke prevention in atrial fibrillation in patients without a severe impairment of the renal function. In the RELY-trial, the vast majority of peak plasma concentrations measured 2 h after drug intake in both the 110 mg bid and the 150 mg bid regimen were below 400 ng/ml [18]. Comparing the DE plasma concentrations achieved in the two dose groups of our study (253.3±30.6 ng/ml in the group receiving 37.5 mg/kg and 431.1±69.8 ng/ml in the group receiving 75 mg/kg) to these clinical data, the lower of the two dose groups represents average peak plasma concentrations reached in human trial subjects taking DE 150 mg bid whereas the higher dose group represents supratherapeutic DE plasma concentrations which may be reached in patients at risk for DE accumulation e. g. due to renal insufficiency. Single DE concentration measurements 72 h after tMCAO also showed supratherapeutic DE levels of 646.0 and 767.2 ng/ml.

We have previously demonstrated that warfarin pretreatment with INR values in the therapeutic range used in humans (INR 2–3) leads to a considerable increase in the risk of HT in a similar model of large territorial cerebral infarctions [7]. Contrary to those findings, we did not see any significant increase in HT in mice subjected to tMCAO under oral anticoagulation with DE, even when DE anticoagulation to supratherapeutic levels was continued for 72 h. From a translational point of view, these findings suggest that the risk HT in patients suffering a stroke under oral anticoagulation with DE might be less problematic than in patients anticoagulated with warfarin and comparable to non-anticoagulated patients and hence it might be safe to continue oral anticoagulation with DE after stroke.

Whereas vitamin K antagonists like warfarin reduce the plasma concentration of coagulation factors II, VII, IX and X, the direct thrombin inhibitor dabigatran only targets factor II, resulting in a less important impact on the coagulation cascade. Previous studies have shown that deficiencies of the coagulation factors II, VII and X cause delayed clot initiation and affect clot propagation and clot strength [20]. Anyhow, a small amount of factor II seems to be enough for similar clot initiation values as in control plasma. By contrast, almost every decrease of the coagulation factors VII and X prolongs time to clot [20]. Interestingly, in the RE-LY trial, DE anticoagulation specifically reduced the risk of intracerebral hemorrhage compared to warfarin both in the 110 mg bid and in the 150 mg bid group in patients aged younger and older than 75 years whereas there was a trend towards a higher rate of all major bleedings in the patients over 75 years receiving DE 150 mg bid compared to the warfarin group [21]. To date, the mechanism of this specific protection from intracerebral hemorrhagic complications under DE treatment compared to warfarin is not clear. It has been hypothesized that the tissue factor (TF)/factor VIIa interaction, that provides additional protection in the brain because the brain shows a high TF expression, is disrupted in warfarin-treated patients due to the blockage of vitamin K-dependent γ-carboxylation [22]. In DE treated patients, interaction between TF, which is a transmembrane receptor for factor VIIa, and factor VIIa is not impaired.

Another important characteristic of DE should be mentioned in this context. The thrombin molecule has three points of interest for interaction with direct thrombin inhibitors, the active enzyme site and two exosites, one for fibrin binding and one for heparin binding [23], [24]. Traditional anticoagulants such as unfractionated heparin and low-molecular-weight heparin need antithrombin as a cofactor, whereas the univalent thrombin inhibitor DE binds directly at the active site of thrombin. The exosite 1 is still free for fibrin polymerization by bridging between fibrinogen molecules [22]. This might be another reason for the small amount of HT in our study. Consistently, data from a recent study of our group showed that DE pretreatment does not lead to hematoma enlargement in two different models of intracerebral hemorrhage in mice, whereas the bivalent direct thrombin inhibitor lepirudin leads to hematoma growth comparable to heparin or warfarin pretreatment [12].

Some important shortcomings of the present study should be mentioned. First, even though the coagulation systems of mice and men are largely similar both in their physiologic properties and in their reactions in coagulation assays, the differences between the murine and human coagulation system have not been assessed with regard to the reaction to DE treatment [25]. Another point of criticism might be that we only used proximal MCA occlusion with the filament model leading to territorial infarctions. This type of infarctions represents one end of the broad spectrum of cerebral ischemia. In order not to underestimate the influence of DE pretreatment on spontaneous HT after stroke, we chose moderate to severe infarctions and a high-dose DE administration reaching supratherapeutic concentrations. Another issue is the untimely death of a relevant percentage of our animals. Since we planned an intention-to-treat analysis, they are included in the analysis but we tried to make the actual sample sizes of surviving mice very clear.

In summary, our results suggest that DE, which has been shown to have a superior risk-to-benefit profile compared to warfarin for stroke prevention in patients with atrial fibrillation, not only may lead to considerably less HT if an ischemic stroke occurs under anticoagulation but might also be safely continued without interruption after a stroke under anticoagulant treatment. Since a subgroup analysis of the RE-LY trial identified the CHADS_2_ score as a suitable predictor of the intracranial bleeding risk for patients with atrial fibrillation receiving DE [26], the continuation of DE after a stroke might be especially safe in patients with a low CHADS_2_ score. However, direct clinical conclusion from our data obtained in an experimental stroke model in mice should be treated with utmost caution and clinical data on these questions are warranted.

## Supporting Information

Table S1 **14 point neurological deficit score (mNSS).**
(DOCX)

Video S1 **Exemplary video of an mNSS evaluation.**
(MP4)
