# Supplementary material for: No Influence of Dabigatran Anticoagulation on Hemorrhagic Transformation in an Experimental Model of Ischemic Stroke
Source: PLoS One. 2012 Jul 24;7(7):e40804. doi: 10.1371/journal.pone.0040804 (PMC3404053; doi:10.1371/journal.pone.0040804)
Supplement: Table S1 — 14 point neurological deficit score (mNSS). (DOCX) [file pone.0040804.s001.docx]

****Supplemental Table 1****

| 14 point neurological deficit score (mNSS), modified from Chen et al. 2001 [13] | |
| --- | --- |
| **Extent of hemiparesis (max. 3 points)**  Test: Raising the mouse by the tail | 1 Flexion of forelimb  1 Flexion of hindlimb  1 Head moved more than 10 ° to the vertical axis  within 30 seconds |
| **Gait (max. 3 points)**  Test: Allow the mouse to walk on an even surface | 0 Normal walk  1 Inability to walk straight  2 Circling towards the paretic side  3 Falling towards the paretic side |
| **Coordination (max. 6 points)**  Test: Allow the mouse to balance on a round beam of 8 mm diameter | 0 Mouse balances with steady posture  1 Mouse grasps side of beam  2 Mouse hugs the beam and one limb falls down  from the beam  3 Mouse hugs the beam and two limbs fall down  from the beam, or spins on beam (>30  seconds)  4 Mouse attempts to balance on the beam but  falls off (>20 seconds)   5 Mouse attempts to balance on the beam but  falls off (>10 seconds)  6 Mouse falls off: No attempt to balance or hang  on to the beam (<10 seconds) |
| Sensory function (max. 2)  Test: Corneal reflex, Pinna reflex (head shake upon touch of the external auditory meatus) | 1 Absence of corneal reflex  2 Absence of Pinna reflex |
